# Association Between Post-COVID Consequences and Cognitive Performance in University Students: A Cross-Sectional Study

**DOI:** 10.3390/ejihpe16090131

**Published:** 2026-08-30

**Authors:** Sergey Malykh, Valeriia Demareva, Victoria Ismatullina, Timofey Adamovich, Tamara Malykh, Tatiana Tikhomirova

**Affiliations:** 1 Federal State Budget Scientific Institution “Federal Scientific Center of Psychological and Multidisciplinary Research”, 125009 Moscow, Russia; 2 Cyberpsychology Department, Faculty of Social Sciences, Lobachevsky State University of Nizhny Novgorod, 603950 Nizhny Novgorod, Russia; valeriia.demareva@fsn.unn.ru

**Keywords:** post-COVID consequences, COVID-19, visuospatial working memory, choice-response accuracy, cognitive performance, university students, young adults

## Abstract

Post-COVID symptoms frequently include cognitive complaints, yet evidence on objectively measured cognitive performance in large samples of young adults remains limited. This study examined associations between self-reported post-COVID consequences and objective cognitive performance in university students, with particular attention to acute COVID-19 severity. The sample comprised 7482 students aged 17–23 years: 3945 reported no previous COVID-19, 1676 reported previous COVID-19 without post-COVID consequences, and 1861 reported previous COVID-19 with post-COVID consequences. Visuospatial working memory was assessed using the Corsi Block Tapping Test (CTB), and choice-response performance was assessed using accuracy and mean correct-response latency in a four-choice reaction time task. Analyses accounted for age, sex, and clustering by university; among previously infected participants, models additionally adjusted for perceived acute COVID-19 severity. The primary adjusted analyses included 7117 participants for CTB and 6361 participants for the cleaned choice-response accuracy and latency analyses. In the three-group analysis, students reporting post-COVID consequences had slightly lower CTB scores than those without previous COVID-19 (B = −0.146, 95% CI [−0.277, −0.015], *p* = 0.029), although the effect was small and was not fully robust in an ordinal sensitivity model (OR = 0.91, 95% CI [0.82, 1.00], *p* = 0.052). No reduction in choice-response accuracy or robust slowing of response latency was observed in the post-COVID consequence group. Among previously infected participants, post-COVID consequences were not significantly associated with CTB performance (B = −0.149, 95% CI [−0.311, 0.013], *p* = 0.071), choice-response accuracy (OR = 0.80, 95% CI [0.60, 1.07], *p* = 0.126), or response latency (−0.79%, 95% CI [−2.06%, 0.49%], *p* = 0.224) after adjustment for acute severity, age, and sex; none of these associations remained significant after false discovery rate correction. Acute COVID-19 severity was likewise not significantly associated with the cognitive outcomes. Overall, self-reported post-COVID consequences were associated with, at most, small and outcome-dependent differences in objective cognitive performance in this young university sample. The findings do not support a broad objective cognitive deficit associated with self-reported post-COVID consequences after accounting for acute disease severity and demographic factors.

## 1. Introduction

Since the COVID-19 pandemic, increasing attention has been directed toward health problems that persist beyond the acute phase of infection. The World Health Organization defines post-COVID condition as symptoms that usually occur within three months of the onset of COVID-19, persist for at least two months, and cannot be explained by an alternative diagnosis (World Health Organization, 2021). Commonly reported manifestations include fatigue, sleep disturbances, emotional symptoms, respiratory complaints, and cognitive difficulties (Nalbandian et al., 2021). Cognitive complaints, often described as “brain fog,” may involve perceived difficulties with concentration, memory, and mental efficiency and can interfere with everyday activities, work, and education (Ceban et al., 2022; Premraj et al., 2022). Importantly, however, subjective cognitive complaints and objectively measured cognitive performance represent related but distinct phenomena and should not be treated as interchangeable indicators of cognitive impairment.

Evidence accumulated over the past several years suggests that cognitive changes following COVID-19 are not limited to subjective complaints. Differences have been reported across several objectively assessed domains, including working memory, attention, executive functioning, and processing speed (Becker et al., 2021; Crivelli et al., 2022). Population-based investigations have identified measurable cognitive differences even among individuals who experienced relatively mild disease and did not require hospitalization (Hampshire et al., 2021). Neuroimaging findings from the UK Biobank have also demonstrated structural brain changes accompanied by measurable cognitive decline following SARS-CoV-2 infection (Douaud et al., 2022). More recent evidence indicates that individuals reporting persistent post-COVID symptoms may show poorer objective cognitive performance than those who recover without continuing symptoms (Hampshire et al., 2024). At the same time, reported effects vary across cognitive domains, samples, and assessment methods, and statistically detectable group differences do not necessarily indicate clinically meaningful impairment.

The mechanisms underlying post-COVID cognitive difficulties remain incompletely understood. Proposed pathways include persistent neuroinflammation, endothelial dysfunction, immune dysregulation, and microvascular abnormalities (Yong, 2021; Taquet et al., 2021; Oronsky et al., 2023). Cognitive performance may also be associated with fatigue, sleep disturbance, anxiety, and depressive symptoms that frequently accompany post-COVID complaints (Nalbandian et al., 2021; Ziauddeen et al., 2022). Biomarker studies have additionally linked longer-term cognitive deficits with indicators of neuronal injury and structural brain abnormalities (Wood et al., 2025). These findings suggest that post-COVID cognitive outcomes may reflect multiple interacting biological and psychological processes rather than a single mechanism attributable solely to the severity of the acute infection.

Although research on post-COVID cognitive outcomes has expanded rapidly, evidence in young adults remains comparatively limited. Much of the available literature has focused on hospitalized patients, clinical cohorts, or middle-aged and older populations (Ceban et al., 2022; Crivelli et al., 2022). University students are of particular interest because working memory, attention, and efficient responding are directly relevant to learning and academic activities. At the same time, cognitive effects in young, predominantly non-hospitalized populations may be subtle. In large samples, even very small differences can reach statistical significance, whereas brief computerized tasks may also show restricted variability, floor or ceiling effects, or differential sensitivity across cognitive domains. Consequently, both statistical significance and effect magnitude need to be considered when evaluating post-COVID differences in this population.

The distinction between subjective post-COVID complaints and objective cognitive performance is particularly important in the present context. Self-reported post-COVID consequences may include perceived concentration or memory difficulties, but such reports do not establish objective cognitive impairment. Conversely, objective tasks capture specific components of performance rather than the broader experience of cognitive difficulties in everyday life. The present study therefore focuses on two objectively assessed domains: visuospatial working memory measured with the Corsi Block Tapping Test and choice-response performance assessed through accuracy and response latency. These measures provide complementary information about short-term visuospatial information maintenance and relatively simple response performance, while not representing a comprehensive neuropsychological assessment.

Another unresolved issue concerns acute COVID-19 severity. Previous investigations have generally associated more severe acute illness with poorer subsequent cognitive outcomes (Hampshire et al., 2021; Becker et al., 2021). However, persistent post-COVID complaints can also occur following less severe infection, and the extent to which self-reported post-COVID consequences are associated with objective cognitive performance after accounting for perceived acute severity remains uncertain. Examining these factors simultaneously may help distinguish associations with continuing post-COVID complaints from those related to the reported severity of the acute episode.

The present analysis uses data from the same participants and the same assessment session as Malykh et al. (2026), but examines different outcome variables. That publication focused on psychological outcomes, including well-being, subjective happiness, life satisfaction, and trait anxiety. The current study addresses a distinct research question using computerized cognitive outcomes collected within the same assessment framework but not analyzed in the previous report. This distinction is important because the present study evaluates objective task performance rather than the psychological outcomes examined previously.

In the present study, we compared objective cognitive performance among students reporting no previous COVID-19, students reporting previous COVID-19 without post-COVID consequences, and students reporting previous COVID-19 with self-reported post-COVID consequences. We then examined, among previously infected participants, whether post-COVID consequence status was associated with cognitive performance after adjustment for perceived acute COVID-19 severity, age, and sex. Because the available post-COVID measure did not apply the duration and diagnostic criteria required by the WHO clinical definition, the construct examined here is interpreted as self-reported post-COVID consequences rather than clinically defined post-COVID condition.

We hypothesized that students reporting post-COVID consequences would, on average, demonstrate poorer objective cognitive performance than both comparison groups. Based on previous evidence relating acute disease burden to later cognitive outcomes, we further expected greater acute COVID-19 severity to be associated with poorer cognitive performance among previously infected participants. Finally, we expected self-reported post-COVID consequence status to remain associated with cognitive performance after adjustment for demographic characteristics and perceived acute disease severity. Given the expected subtlety of cognitive differences in a young university sample, effect sizes and confidence intervals were considered alongside statistical significance.

## 2. Materials and Methods

### 2.1. Participants

The present study analyzed data from a nationwide multicenter survey involving students from 30 universities across all federal districts of the Russian Federation. Psychological outcomes from this cohort, including well-being, subjective happiness, life satisfaction, and trait anxiety, have been reported previously (Malykh et al., 2026). The present study addresses a distinct research question by examining objective cognitive performance, which was not analyzed in the previous publication.

The initial sample comprised 7482 university students aged 17–23 years (M = 18.51, SD = 0.96), including 2694 males (36.0%) and 4788 females (64.0%). Sex was coded as 1 = male and 2 = female. Because cognitive outcomes were analyzed using available-case data, the number of participants varied across individual analyses according to outcome availability.

Based on self-reported COVID-19 history, participants were classified into three groups:

- No previous COVID-19 infection (*n* = 3945);
- Previous COVID-19 without self-reported post-COVID consequences (*n* = 1676);
- Previous COVID-19 with self-reported post-COVID consequences (*n* = 1861).

Participants who reported previous COVID-19 infection (*n* = 3537) also rated the severity of their acute illness on a four-level ordinal scale, with higher values indicating greater perceived severity.

University affiliation for multilevel analyses was reconstructed from institution-specific prefixes embedded in anonymized participant identifiers. An institution-specific code could be identified for 7378 of the 7482 participants (98.6%). Participants for whom university affiliation could not be reconstructed were retained in descriptive analyses but were excluded from models requiring university-level clustering. Depending on cognitive-outcome availability, the multilevel analytic samples comprised participants from 21–22 identifiable university clusters.

The study complied with the Declaration of Helsinki and was approved by the Ethics Committee of the Psychological Institute of the Russian Academy of Education (Protocol No. 2020/4-1, 2 April 2020). Written informed consent was obtained from all participants. For respondents younger than 18 years, parental consent and participant assent were additionally obtained.

### 2.2. Measures

#### 2.2.1. COVID-19 Variables

Participants indicated whether they had previously experienced COVID-19. Those reporting infection were additionally asked whether they experienced post-COVID consequences. Reported consequences included concentration difficulties, memory problems, increased anxiety, decreased mood, and other ongoing symptoms.

From these responses, a binary indicator of post-COVID consequences was created (0 = absent; 1 = present). This variable was subsequently combined with COVID-19 history to generate the three-group classification used throughout the analyses.

Participants with previous COVID-19 also reported the perceived severity of the acute infection using a four-category ordinal scale, which served as an indicator of acute disease burden in subsequent analyses. The original verbal labels associated with the four severity categories were not retained in the analytic dataset available for the present study; acute severity was therefore treated as a self-reported ordinal indicator rather than as a clinically validated measure of disease severity.

The post-COVID consequence variable did not incorporate a minimum symptom-duration criterion and therefore should not be interpreted as equivalent to a clinically diagnosed post-COVID condition. Information on time since infection, vaccination status, reinfection, and clinical confirmation of infection was not available in the analytic dataset. Accordingly, throughout the manuscript the construct is referred to as self-reported post-COVID consequences rather than clinically diagnosed post-COVID condition.

#### 2.2.2. Corsi Block Tapping Test

Visuospatial working memory was assessed using a computerized version of the Corsi Block Tapping Test (CTB) (Corsi, 1972; Tikhomirova et al., 2020).

The task consisted of nine fixed blocks displayed on the screen. During each trial, a sequence of highlighted blocks was presented (1 s per block with 1 s intervals), after which participants reproduced the sequence by clicking the blocks in the same order. Before beginning the task, participants completed a repeatable three-block practice trial with feedback.

The main test consisted of 12 trials (two trials for each sequence length from four to nine blocks) presented in ascending order. Testing was terminated when both trials of the same sequence length were answered incorrectly. Each correctly reproduced sequence was scored as one point, producing a total score ranging from 0 to 12. In addition to the total score, several auxiliary indices (task duration, response speed, and number of completed trials) were recorded but were not included in the present analyses.

#### 2.2.3. Choice Reaction Time Task

Information-processing performance was assessed using a computerized four-choice reaction time task (Tikhomirova et al., 2020). Two distinct outcomes from this task were analyzed in the present study: choice-response accuracy and mean correct-response latency.

Digits from 1 to 4 were presented individually at the center of the screen, and participants responded by pressing the corresponding keyboard key as quickly and accurately as possible. The task consisted of 40 experimental trials, with each digit presented ten times in random order. Each stimulus remained visible for a maximum of 8 s or until a response was made.

Before the experimental trials, participants completed six practice trials with accuracy feedback. During the main task, no feedback was provided. For each trial, response accuracy and reaction time were recorded. Choice-response accuracy was defined as the total number of correct responses across the 40 experimental trials and therefore ranged from 0 to 40, with higher scores indicating better performance. Mean correct-response latency was calculated from correct responses and expressed in milliseconds, with lower values indicating faster responding. The task required approximately four minutes to complete.

### 2.3. Procedure

Data collection was conducted in university computer classrooms using a standardized testing protocol. Assessments were supervised by trained research personnel.

Participants first completed demographic and COVID-19 questionnaires, followed by the computerized cognitive tasks. Completion of the entire assessment required approximately 15–20 min. The exact individual data-collection dates and the overall collection window were not retained in the analytic dataset available for the present study. Accordingly, the timing of assessment relative to infection and the pandemic period in which individual infections occurred could not be reconstructed.

### 2.4. Statistical Analysis

All statistical analyses were performed in R version 4.4.1 (R Core Team, 2024). Descriptive statistics were calculated for demographic characteristics and cognitive outcomes. Continuous variables are reported as means and standard deviations or medians and interquartile ranges where appropriate, and categorical variables as frequencies and percentages.

Linear mixed-effects models were fitted using lmer from the lme4 package with inferential procedures from lmerTest; cumulative-link mixed models were fitted using clmm from ordinal; CR2 cluster-robust inference was obtained using clubSandwich; and Games–Howell comparisons were performed using rstatix.

Unadjusted descriptive comparisons across the three groups defined by COVID-19 history and self-reported post-COVID consequences were initially examined using Welch’s one-way ANOVA with Games–Howell post hoc comparisons because homogeneity-of-variance tests indicated unequal variances across groups. These analyses were treated as descriptive; the primary inferential analyses accounted for age, sex, and university clustering. Effect sizes for CTB group contrasts were additionally expressed as Cohen’s d with 95% confidence intervals.

Because participants were recruited from multiple universities and cognitive performance showed non-negligible between-university variation, primary adjusted analyses accounted for clustering by university. University affiliation was reconstructed from institution-specific prefixes in anonymized participant identifiers, as described above. Twenty-two university clusters were represented in the CTB analytic sample and 21 in the choice-response analyses.

For the Corsi Block Tapping Test, adjusted group differences were estimated using linear mixed-effects models with a random intercept for university. COVID-19 group, age, and sex were included as fixed effects. The intraclass correlation coefficient (ICC) was calculated from the unconditional model to quantify between-university variance. Because CTB scores were discrete and bounded between 0 and 12, a cumulative-link mixed model was additionally fitted as a sensitivity analysis. Floor and ceiling frequencies were examined descriptively to assess the distribution of the bounded CTB score.

The choice reaction time task yielded two distinct outcomes: response accuracy, defined as the number of correct responses out of 40 trials, and mean response latency for correct responses. Accuracy scores showed a pronounced ceiling effect and substantial negative skew; distributional diagnostics therefore included the proportion of participants attaining the maximum score. Accuracy was modeled as a binomial count of correct and incorrect responses. Because the binomial model showed substantial overdispersion, quasibinomial regression was used, with cluster-robust CR2 standard errors and Satterthwaite-adjusted degrees of freedom to account for clustering by university. To reduce potential speed–accuracy confounding, the primary accuracy model additionally included log-transformed mean correct-response latency as a covariate and was restricted to participants with plausible mean correct-response latencies of at least 300 ms.

Mean correct-response latency was analyzed separately. Four participants had a recorded mean correct-response latency of 0 ms; all four had zero correct responses, indicating that these values could not represent valid reaction times. These observations were therefore excluded. Because the latency distribution was right-skewed, reaction times were log-transformed and analyzed using linear mixed-effects models with a random intercept for university. In the primary cleaned latency analysis, mean correct-response latencies below 300 ms were additionally excluded as implausibly fast for the present task. As a sensitivity analysis addressing the opposite tail of the distribution, observations above the 99th percentile were excluded. Regression coefficients from log-latency models were transformed to percentage differences using 100 × [exp(B) − 1].

To examine whether self-reported post-COVID consequences were associated with cognitive performance after accounting for perceived acute COVID-19 severity, analyses were repeated in the subsample of participants reporting previous COVID-19. Models simultaneously included post-COVID consequence status, acute COVID-19 severity, age, and sex. Acute COVID-19 severity was entered as a categorical predictor, with the lowest severity category serving as the reference category. For CTB and response latency, university was modeled as a random intercept. For response accuracy, quasibinomial regression with CR2 cluster-robust inference by university was used. Unlike the primary three-group accuracy analysis, this infected-only model did not include response latency as a covariate and was therefore not restricted to participants with mean correct-response latencies ≥300 ms. This specification was used to retain the largest available infected subsample for the severity-adjusted accuracy analysis, while response latency was examined separately as an outcome.

Potential effect modification by acute COVID-19 severity was additionally examined by including severity × post-COVID consequence interaction terms.

Because trait anxiety was available for this cohort and may be related to both persistent symptom reporting and cognitive performance, an additional sensitivity analysis was conducted for CTB in the previously infected subsample. This model included trait anxiety in addition to post-COVID consequence status, acute COVID-19 severity, age, sex, and the random intercept for university.

Missingness was evaluated separately for the CTB and choice-response task. CTB missingness was modeled using logistic regression with COVID-19 group, age, and sex as predictors. For choice-response task non-completion, logistic regression additionally included available CTB performance to assess whether missingness was associated with cognitive performance. No missing values were imputed; analyses were based on participants with complete data for the variables required by each corresponding model.

To address multiple testing across the three prespecified cognitive outcomes in the previously infected subsample—CTB performance, choice-response accuracy, and response latency—the *p* values for the post-COVID consequence term were adjusted using the Benjamini–Hochberg false discovery rate procedure. Two-sided *p* values below 0.05 were considered statistically significant. All effect estimates are reported with 95% confidence intervals.

## 3. Results

### 3.1. Sample Characteristics and Availability of Cognitive Data

The final dataset included 7482 participants: 3945 participants who reported no history of COVID-19, 1676 who reported previous COVID-19 without self-reported post-COVID consequences, and 1861 who reported previous COVID-19 with self-reported post-COVID consequences.

Corsi Block Tapping Test (CTB) scores were available for 7211 participants (96.4% of the total sample). Missingness was similar across the three COVID-19 groups: 3.57% in the no-COVID group, 3.94% in the COVID-without-consequences group, and 3.44% in the COVID-with-consequences group.

Choice-response task data were available for 6384 participants (85.3%). All 271 participants with missing CTB scores also lacked choice-response data, whereas an additional 827 participants had CTB scores but no choice-response data.

CTB missingness was not associated with COVID-19 group. Relative to participants without previous COVID-19, the odds of CTB missingness did not differ for participants with COVID-19 without consequences (OR = 1.09, 95% CI [0.81, 1.47], *p* = 0.558) or for those reporting post-COVID consequences (OR = 1.01, 95% CI [0.74, 1.36], *p* = 0.960). Missingness was somewhat more frequent with increasing age (OR = 1.17, 95% CI [1.05, 1.30], *p* = 0.004) and differed by sex (OR = 0.73, 95% CI [0.57, 0.94], *p* = 0.013). See Table 1.

Choice-response task non-completion differed across COVID-19 groups, occurring in 13.0% of participants without previous COVID-19, 14.9% of those with previous COVID-19 without post-COVID consequences, and 18.1% of those reporting post-COVID consequences. In the adjusted logistic model, non-completion was more likely among participants with COVID-19 without consequences (OR = 1.24, 95% CI [1.02, 1.50], *p* = 0.026) and among those with post-COVID consequences (OR = 1.49, 95% CI [1.25, 1.76], *p* < 0.001) than among participants without previous COVID-19. Choice-response non-completion was also associated with age (OR = 0.84, 95% CI [0.77, 0.91], *p* < 0.001), sex (OR = 2.22, 95% CI [1.86, 2.67], *p* < 0.001), and lower available CTB performance (OR = 0.89 per CTB point, 95% CI [0.86, 0.92], *p* < 0.001).

### 3.2. Cognitive Performance Across the Three COVID-19 Groups

#### 3.2.1. Visuospatial Working Memory

CTB scores covered the full 0–12 range. A total of 251 participants (3.48% of those with CTB data) scored 0, whereas 54 participants (0.75%) attained the maximum score of 12, indicating a modest floor frequency and no substantial ceiling effect.

The primary multilevel CTB analysis included 7117 participants from 22 universities. After adjustment for age and sex and inclusion of a random intercept for university, previous COVID-19 without self-reported post-COVID consequences was not associated with CTB performance (B = 0.058, 95% CI [−0.077, 0.193], *p* = 0.400). Participants reporting post-COVID consequences showed slightly lower CTB scores than participants without previous COVID-19 (B = −0.146, 95% CI [−0.277, −0.015], *p* = 0.029). However, the standardized magnitude of this difference was small (standardized B = −0.062, 95% CI [−0.119, −0.006]).

Unadjusted standardized contrasts were similarly small. The difference between the no-COVID and COVID-with-consequences groups corresponded to Cohen’s d = 0.09 (95% CI [0.03, 0.14]), while the contrast between the two previously infected groups corresponded to d = 0.14 (95% CI [0.07, 0.21]).

Because CTB scores were discrete and bounded, the analysis was repeated using a cumulative-link mixed model. In this model, COVID-19 without consequences was not associated with CTB performance (OR = 1.07, 95% CI [0.96, 1.18], *p* = 0.216). The association for COVID-19 with consequences was also small and did not reach the conventional significance threshold (OR = 0.91, 95% CI [0.82, 1.00], *p* = 0.052).

Thus, the small CTB difference observed in the linear mixed model was not fully robust to alternative modeling of the bounded outcome.

#### 3.2.2. Choice-Response Accuracy

Choice-response accuracy showed a pronounced ceiling effect, with participants completing approximately 38 of 40 trials correctly on average. Accuracy was therefore modeled as a binomial count of correct and incorrect responses, with quasibinomial estimation used to address overdispersion and CR2 cluster-robust inference used to account for university clustering.

In the analysis additionally accounting for response latency and restricted to participants with plausible mean correct-response latencies (≥300 ms; *n* = 6361 from 21 universities), the COVID-without-consequences group showed slightly higher accuracy than the no-COVID group (OR = 1.17, 95% CI [1.06, 1.29], *p* = 0.006). In contrast, participants reporting post-COVID consequences did not differ from the no-COVID group (OR = 0.97, 95% CI [0.78, 1.20], *p* = 0.729).

Mean response latency itself was not significantly associated with accuracy (OR = 0.85, 95% CI [0.31, 2.36], *p* = 0.738).

These results provided no evidence of reduced choice-response accuracy specifically associated with post-COVID consequences.

#### 3.2.3. Response Latency

Mean correct-response latency was initially available for 6384 participants. Four observations had a recorded value of 0 ms; all four participants had zero correct responses, indicating that these values did not represent valid reaction times.

After their exclusion, the latency analysis included 6,380 participants from 21 universities. Mean latency was 620 ms (SD = 138), and median latency was 595 ms. The unconditional multilevel model yielded an ICC of 0.105, indicating that 10.5% of variance in log-transformed latency occurred between universities.

In the initial multilevel model, previous COVID-19 without consequences was unrelated to response latency (−0.03%, *p* = 0.957). Participants reporting post-COVID consequences showed slightly shorter latencies than participants without previous COVID-19 (−1.38%, 95% CI [−2.60%, −0.14%], *p* = 0.030).

However, 19 participants had implausibly fast positive mean correct-response latencies below 300 ms. After excluding these observations, the difference associated with post-COVID consequences was attenuated to −0.46% and was no longer statistically significant (*p* = 0.394). The COVID-without-consequences group likewise did not differ from the reference group (−0.10%, *p* = 0.850).

Exclusion of values above the 99th percentile produced an estimate of −1.24% (95% CI [−2.39%, −0.07%], *p* = 0.038). Thus, the very small latency difference depended on the treatment of extreme observations and did not constitute robust evidence of altered response speed. See Table 2.

### 3.3. Post-COVID Consequences Among Previously Infected Participants

The three-group analyses do not fully distinguish self-reported post-COVID consequences from characteristics of the acute infection. Therefore, the principal specificity analysis was restricted to participants reporting previous COVID-19 and simultaneously modeled post-COVID consequence status and acute COVID-19 severity.

There was no evidence that the association between post-COVID consequence status and cognitive performance differed across acute-severity categories. No significant severity × post-COVID consequence interaction was observed for CTB (F = 1.12, *p* = 0.341), choice-response accuracy (F = 1.30, *p* = 0.271), or response latency (F = 0.05, *p* = 0.987).

Post-COVID consequence prevalence varied across the four acute-severity categories (77.9%, 73.5%, 42.6%, and 60.0% for categories 1–4, respectively). Generalized variance inflation factors indicated no problematic multicollinearity between acute severity and post-COVID consequence status (GVIF-adjusted values approximately 1.01 and 1.06, respectively).

#### 3.3.1. Visuospatial Working Memory

The CTB model included 3346 previously infected participants from 22 universities. None of the acute COVID-19 severity categories differed significantly from the reference severity category (all *p* ≥ 0.067). This conclusion was unchanged in sensitivity analyses treating severity as an ordinal linear predictor, changing the reference category, and excluding the smallest severity category.

After adjustment for acute severity, age, sex, and university, post-COVID consequences were associated with an estimated 0.149-point lower CTB score, but the confidence interval included zero (B = −0.149, 95% CI [−0.311, 0.013], *p* = 0.071).

#### 3.3.2. Choice-Response Accuracy

For choice-response accuracy, neither acute COVID-19 severity nor post-COVID consequence status was significantly associated with performance under CR2 cluster-robust inference.

For post-COVID consequences, B = −0.222 (95% CI [−0.515, 0.072], *p* = 0.126), corresponding to OR = 0.80 (95% CI [0.60, 1.07]).

#### 3.3.3. Response Latency

The latency analysis included 2928 previously infected participants from 21 universities after exclusion of implausibly fast mean latencies.

None of the acute severity categories was significantly associated with latency. Post-COVID consequences were associated with an estimated −0.79% difference in response latency (95% CI [−2.06%, 0.49%], *p* = 0.224).

After Benjamini–Hochberg correction across the three cognitive outcomes, none of the associations with post-COVID consequences remained statistically significant: pFDR = 0.188 for CTB, 0.188 for choice-response accuracy, and 0.224 for response latency. See Table 3.

**Figure 1:**
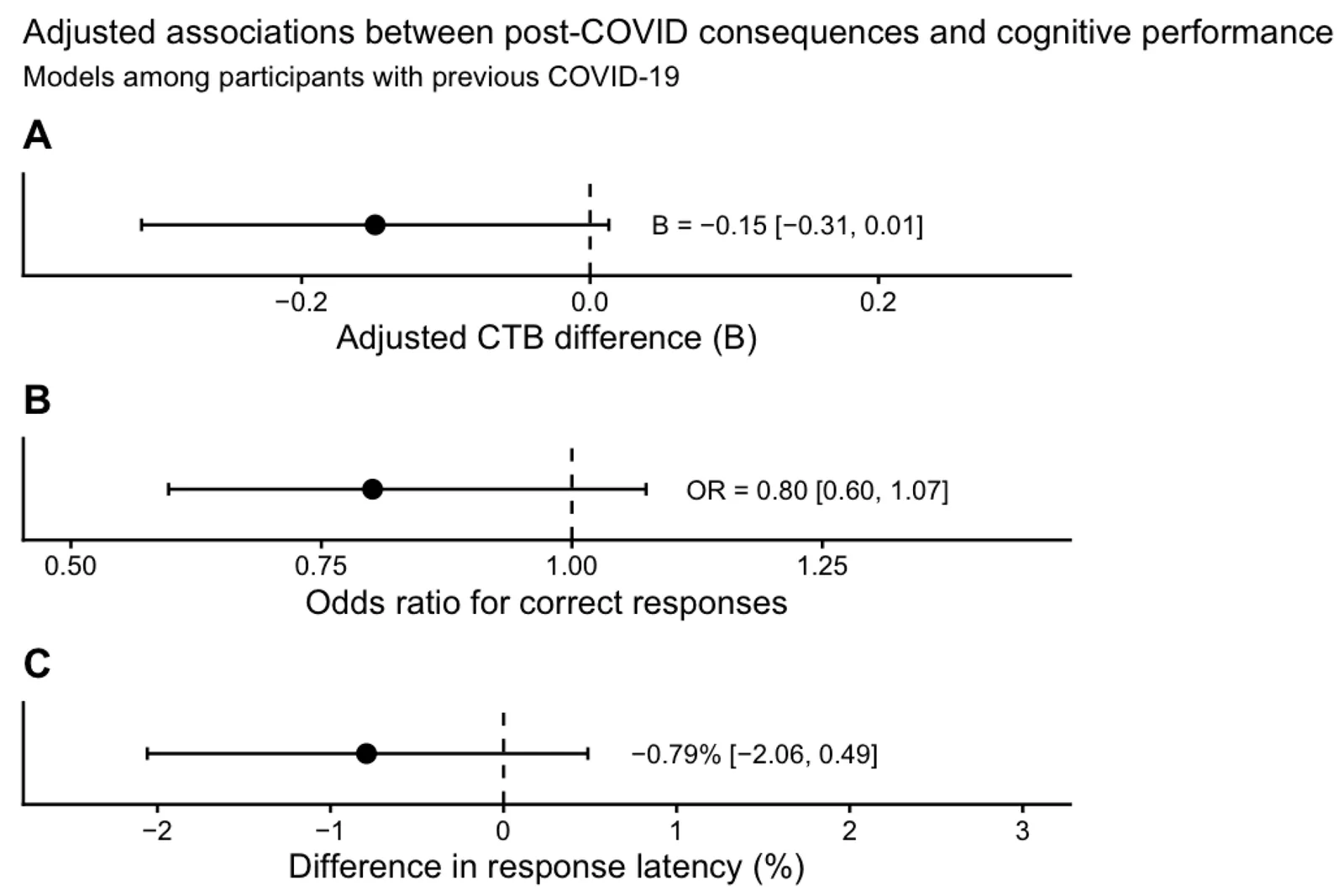
Adjusted associations between self-reported post-COVID consequences and cognitive performance among participants with previous COVID-19: (**A**) CTB performance; (**B**) choice-response accuracy; (**C**) response latency. Points represent adjusted effect estimates and horizontal lines represent 95% confidence intervals. Models were adjusted for acute COVID-19 severity, age, and sex and accounted for clustering by university. CTB is presented as an unstandardized regression coefficient, choice-response accuracy as an odds ratio, and response latency as the percentage difference in mean correct-response latency.

To facilitate comparison across cognitive outcomes, the adjusted associations between post-COVID consequences and CTB performance, choice-response accuracy, and response latency are summarized visually in Figure 1. Although the three outcomes are expressed on different effect scales, the figure provides an overview of the direction, magnitude, and uncertainty of the post-COVID consequence estimates across the cognitive measures.

### 3.4. Additional Adjustment for Trait Anxiety

Because trait anxiety may be related both to persistent symptom reporting and cognitive performance, an additional sensitivity analysis was conducted for CTB.

The model included 3,310 previously infected participants from 22 universities. After adjustment for trait anxiety, the association between post-COVID consequences and CTB performance was attenuated from B = −0.149 to B = −0.099 (95% CI [−0.264, 0.067], *p* = 0.243).

Trait anxiety itself was associated with slightly lower CTB performance (B = −0.0133 per scale point, 95% CI [−0.0207, −0.0058], *p* < 0.001). Acute COVID-19 severity remained unrelated to CTB performance.

The estimated association between post-COVID consequence status and CTB performance was smaller after additional adjustment for trait anxiety, while trait anxiety itself was independently associated with slightly lower CTB scores. See Table 4.

## 4. Discussion

The present study examined whether self-reported post-COVID consequences were associated with objective cognitive performance in a large multicenter sample of university students. Overall, the findings provide limited evidence for broad cognitive differences associated with post-COVID consequence status in this young population. A small difference in visuospatial working-memory performance was observed in the three-group linear mixed model, but its magnitude was very small and the association did not fully replicate when CTB scores were modeled as an ordinal outcome. Choice-response accuracy did not show poorer performance in students reporting post-COVID consequences, and the small difference initially observed in response latency was sensitive to the treatment of implausibly fast observations. Most importantly, among previously infected participants, post-COVID consequence status was not significantly associated with any of the three cognitive outcomes after simultaneous adjustment for perceived acute COVID-19 severity, age, sex, and university clustering, and none of the associations survived false discovery rate correction.

These results suggest that self-reported post-COVID consequences in university students should not be interpreted as indicating a generalized objective cognitive deficit. Instead, any differences appear to be small, outcome-dependent, and sensitive to analytic specification. This distinction is particularly important because the post-COVID classification used in the present study was based on self-reported consequences rather than a clinically verified post-COVID condition and included subjective concentration and memory complaints among the possible consequences.

### 4.1. Post-COVID Consequences and Cognitive Performance

The present findings complement our earlier analysis of the same student cohort, in which self-reported post-COVID consequences were associated with poorer psychological well-being and higher trait anxiety (Malykh et al., 2026). The current study examined different outcomes collected within the same broader assessment framework, namely performance on computerized cognitive tasks. Taken together, the two analyses indicate that self-reported post-COVID consequences may be more clearly reflected in subjective psychological outcomes than in broad and consistently detectable differences in objective cognitive performance. Because both analyses are cross-sectional, neither can establish whether the reported post-COVID consequences preceded, followed, or contributed causally to the observed psychological or cognitive characteristics.

The clearest cognitive difference in the present study concerned visuospatial working memory. In the primary three-group linear mixed model, students reporting post-COVID consequences had slightly lower CTB scores than students without a reported history of COVID-19. However, the standardized effect was very small, and the corresponding association was attenuated in the cumulative-link mixed model, where it narrowly failed to reach the conventional significance threshold. Moreover, within the previously infected subsample, post-COVID consequence status was no longer significantly associated with CTB performance after adjustment for acute severity, age, sex, and university clustering.

Previous studies have reported cognitive differences following COVID-19, including effects on working memory and related cognitive domains (Hampshire et al., 2021, 2024). Our findings are compatible with the possibility of subtle working-memory differences, but they do not support a strong or generalized impairment in this university sample. This qualification is important because statistical significance in a sample of several thousand participants can arise for effects whose magnitude is very small.

Recent evidence from university populations has also suggested poorer cognitive performance among students with post-COVID condition. Takács et al. (2025), for example, reported differences in verbal working memory. The present study assessed visuospatial rather than verbal working memory, and the correspondence between the two findings should therefore not be overstated. In addition, the CTB effect observed here was small and not fully robust across modeling approaches. Thus, the studies converge in suggesting that working-memory processes may warrant attention after COVID-19, while differing in the specific cognitive domain assessed and in the strength of the observed effects.

The choice-response findings were even less supportive of a post-COVID deficit. Students reporting post-COVID consequences did not show lower accuracy than participants without previous COVID-19 after adjustment for response speed, age, sex, and university clustering. The group reporting previous COVID-19 without consequences actually showed slightly higher accuracy than the no-COVID group, although this difference was also small. The accuracy outcome was characterized by a pronounced ceiling effect, with many participants performing near the maximum score, which necessarily limited its ability to discriminate between individuals with generally high performance.

The latency results likewise require caution. The initial model suggested slightly faster, rather than slower, responding among participants reporting post-COVID consequences. This difference disappeared when implausibly fast mean response times were excluded, whereas exclusion of the upper 1% of the latency distribution yielded a small significant difference again. The lack of stability across reasonable data-cleaning decisions indicates that the latency finding should not be interpreted as evidence for either impaired or enhanced processing speed. Previous studies have similarly shown that cognitive effects after COVID-19 may differ substantially across domains and task characteristics (Becker et al., 2021; Graham et al., 2021).

### 4.2. The Role of Acute COVID-19 Severity

The present findings did not support the hypothesized association between greater acute COVID-19 severity and poorer subsequent cognitive performance. In the primary analyses restricted to previously infected participants, none of the acute-severity categories was significantly associated with CTB performance, choice-response accuracy, or response latency after accounting for post-COVID consequence status and demographic factors.

This result differs from previous studies reporting poorer later cognitive performance after more severe acute COVID-19 (Hampshire et al., 2021; Becker et al., 2021). Several characteristics of the present study may contribute to this discrepancy. First, the sample consisted of young university students rather than predominantly clinical or hospitalized populations, and the range of acute disease severity may have been restricted. Second, acute severity was assessed retrospectively using a self-reported four-category measure rather than clinical indicators. Third, the smallest severity category contained relatively few participants and showed unusually variable choice-response accuracy before removal of extreme scores.

These findings should not be interpreted as evidence that acute disease severity is irrelevant to post-COVID cognition in general. Rather, they indicate that within this particular young university sample and using the available retrospective severity measure, no consistent severity gradient was detected.

### 4.3. Post-COVID Consequences After Accounting for Acute Severity

A central aim of the study was to determine whether self-reported post-COVID consequences were associated with objective cognitive performance after accounting for perceived acute disease severity. The revised analyses provide a more cautious answer than the original models.

Among previously infected students, post-COVID consequence status was associated with a 0.149-point lower CTB score in the adjusted multilevel model, but the confidence interval included zero and the association did not reach the conventional significance threshold. The corresponding associations with choice-response accuracy and latency were likewise non-significant. After correction for multiple testing across the three cognitive outcomes, none of the associations remained statistically significant.

The findings therefore do not support describing post-COVID consequence status as an “independent predictor” of cognitive functioning. At most, they suggest a weak association with visuospatial working-memory performance that becomes uncertain after accounting for acute severity, demographic variables, university clustering, alternative outcome modeling, and multiplicity.

The additional analysis involving trait anxiety further supports this cautious interpretation. Trait anxiety had been measured in the same cohort and was previously shown to be related to post-COVID consequences at the psychological level (Malykh et al., 2026). When trait anxiety was added to the CTB model, the estimated association between post-COVID consequence status and working-memory performance was reduced from B = −0.149 to B = −0.099 and was clearly non-significant, whereas trait anxiety itself was associated with slightly lower CTB scores. This pattern does not establish mediation or causality, but it indicates that the small association between post-COVID consequence status and working-memory performance overlaps with variation in trait anxiety.

This is consistent with the broader view that post-COVID cognitive outcomes may reflect multiple interacting processes rather than a single direct pathway. Persistent biological changes, including neuroinflammatory, vascular, and immune mechanisms, have been proposed as possible contributors to post-COVID cognitive difficulties (Yong, 2021; Taquet et al., 2021), and persistent neurological sequelae have also been described after less severe infection (Al-Aly et al., 2022). At the same time, fatigue, sleep disturbance, anxiety, and depressive symptoms may influence both perceived cognitive difficulties and performance on objective tasks (Nalbandian et al., 2021; Ziauddeen et al., 2022). Biomarker evidence additionally suggests that long-term cognitive outcomes may be associated with ongoing neuronal or structural abnormalities in some populations (Wood et al., 2025). The present cross-sectional data cannot distinguish among these possible pathways.

### 4.4. Sex Differences and Other Covariates

Sex was associated with several cognitive outcomes, although these effects were not the primary focus of the study. In the CTB analyses, female participants tended to obtain slightly lower visuospatial working-memory scores, whereas in some choice-response models they showed higher accuracy. Previous research has reported small sex differences in visuospatial working memory and in speed–accuracy characteristics of response tasks (Voyer et al., 2017; Blough & Slavin, 1987).

These findings should be interpreted cautiously because the study was not designed to test sex differences in cognitive performance. They are nevertheless relevant analytically because the proportion of women differed substantially across COVID-19 groups, particularly in the group reporting post-COVID consequences. Adjustment for sex was therefore important for estimating group differences without conflating post-COVID status with the unequal sex composition of the groups.

Age effects were generally small, which is unsurprising given the restricted age range of 17–23 years. Age adjustment was retained for consistency and because small age associations were observed for some outcomes, but it should not be interpreted as extensive control for developmental variation.

### 4.5. Practical Interpretation

The present findings do not provide a basis for recommending specific academic or clinical interventions on the basis of post-COVID consequence status alone. The observed cognitive differences were very small, varied across outcomes, and were not consistently robust to alternative modeling and sensitivity analyses. In particular, the infected-only analyses did not identify a statistically reliable association between post-COVID consequence status and any of the three objective cognitive outcomes after adjustment and correction for multiple testing.

This does not imply that students reporting persistent difficulties do not require support. Rather, the current data do not demonstrate that self-reported post-COVID consequences identify a group with broad objective cognitive impairment. Future prospective studies should examine whether particular combinations of subjective symptoms, psychological factors, objective cognitive changes, and academic outcomes can identify students who may benefit from targeted support.

### 4.6. Strengths and Limitations

Several strengths of the study should be noted. First, the large multicenter sample provided high statistical power and allowed relatively precise estimation of small effects. Second, the study included both participants reporting previous COVID-19 without post-COVID consequences and participants reporting no previous COVID-19, enabling comparison of post-COVID consequence status with two distinct reference groups. Third, cognitive performance was assessed using computerized tasks rather than inferred exclusively from subjective cognitive complaints. Fourth, the revised analyses explicitly accounted for clustering by university and used outcome-specific statistical approaches to address the bounded CTB distribution, the marked ceiling and overdispersion of choice-response accuracy, and the skewed distribution of response latency.

Several limitations are equally important. First, the cross-sectional design precludes causal conclusions, and no pre-infection cognitive measures were available. It is therefore impossible to determine whether any observed group differences emerged after COVID-19 or were present beforehand. Detailed information on the original sampling frame, procedures used to select participating universities and recruit students within them, the number of students approached, and the response rate was not retained in the analytic materials available for the present study. Consequently, the sample cannot be considered probabilistically representative of Russian university students, and generalization of the findings beyond the participating university sample should be made with caution.

Second, both infection history and post-COVID consequences were based on self-report without serological or other clinical confirmation. Undetected previous infections may therefore have occurred among participants classified as having no history of COVID-19, potentially attenuating between-group differences.

Third, the self-reported post-COVID consequence measure did not include the duration, timing, or diagnostic-exclusion criteria required by the WHO definition of post-COVID condition. Information on time since infection, vaccination status, reinfection, and the pandemic period in which infection occurred was not available. The findings therefore concern self-reported post-COVID consequences and should not be generalized directly to clinically defined post-COVID condition.

Fourth, the consequence classification included subjective concentration and memory difficulties. Consequently, the exposure variable partly incorporated cognitive complaints, although objective task performance and subjective cognitive symptoms are not equivalent. Because the available analytic dataset did not retain the information required to distinguish all clinically meaningful symptom combinations, the present study cannot fully determine whether associations differ between participants with cognitive versus exclusively non-cognitive post-COVID complaints. In addition, expectancy or self-perception effects cannot be excluded: participants who attributed cognitive difficulties to COVID-19 may have approached cognitive testing differently, although the present data do not allow this possibility to be evaluated.

Fifth, residual confounding remains possible. Sleep quality, fatigue, depression, academic stress, physical activity, comorbidities, and socioeconomic factors were not included in the primary models. Trait anxiety was available and was examined in an additional CTB sensitivity analysis; its inclusion attenuated the already small association with post-COVID consequence status. Other unmeasured variables may similarly contribute to both symptom reporting and cognitive performance.

Sixth, missingness was not random with respect to all observed characteristics. Choice-response task non-completion was more frequent among participants reporting post-COVID consequences and was also associated with sex, age, and lower available CTB performance. This pattern raises the possibility of selection bias in the choice-response analyses. In contrast, CTB missingness was low and did not differ materially by COVID-19 group.

Seventh, choice-response accuracy exhibited a strong ceiling effect and substantial overdispersion. Although quasibinomial models and cluster-robust inference were used, and response latency was analyzed separately, the limited variability in accuracy reduced the task’s sensitivity to subtle group differences. Response latency was also sensitive to a small number of implausible extreme observations, reinforcing the need for caution when interpreting very small effects. In addition, the infected-only accuracy model did not adjust for response latency, unlike the primary three-group accuracy model. Therefore, potential speed–accuracy tradeoffs were not explicitly controlled in that specific analysis, although response latency was analyzed separately as an outcome.

Eighth, university affiliation could be reconstructed for most but not all participants, and the identifiable institutional prefixes represented 21–22 clusters in the outcome-specific analyses rather than all 30 universities involved in the broader survey. Nevertheless, accounting for university clustering was important because between-university variation was non-negligible, particularly for response latency.

Finally, the cognitive battery assessed a limited range of functions, primarily visuospatial working memory and relatively simple choice-response performance. These measures should not be interpreted as a comprehensive assessment of cognitive functioning. The sample was also restricted to young university students, limiting generalizability to older adults, clinical populations, or individuals who experienced severe acute COVID-19.

### 4.7. Overall Interpretation

In summary, the revised analyses provide a more qualified picture than would be suggested by statistical significance alone. Self-reported post-COVID consequences were associated with a very small reduction in CTB performance in one primary three-group model, but this finding was not fully robust to alternative modeling and was attenuated after adjustment for acute severity and trait anxiety. Choice-response accuracy did not indicate poorer performance among students reporting post-COVID consequences, and response-latency differences were sensitive to the handling of implausible observations. Acute COVID-19 severity itself showed no consistent association with cognitive performance.

Accordingly, the present findings are most consistent with small, heterogeneous, and outcome-dependent cognitive differences rather than a generalized post-COVID cognitive deficit in university students. Prospective studies incorporating clinically characterized infection history, symptom duration, broader neuropsychological assessment, psychological and behavioral covariates, and longitudinal academic outcomes will be required to determine whether specific subgroups of young adults experience persistent objective cognitive changes following COVID-19.

## 5. Conclusions

In this large multicenter sample of university students, self-reported post-COVID consequences were associated with, at most, small and outcome-dependent differences in objective cognitive performance. A very small reduction in visuospatial working-memory performance was observed in one primary three-group model, but this finding was not fully robust to alternative modeling and was attenuated after additional adjustment. No consistent evidence of reduced choice-response accuracy or altered response latency was found.

Among previously infected participants, self-reported post-COVID consequences were not significantly associated with CTB performance, choice-response accuracy, or response latency after adjustment for perceived acute COVID-19 severity, age, and sex, and none of these associations was statistically significant after false discovery rate correction. Acute COVID-19 severity was likewise not consistently associated with cognitive performance.

These findings do not support a broad objective cognitive deficit associated with self-reported post-COVID consequences in young university students. Rather, any cognitive differences appear to be subtle, heterogeneous, and sensitive to the outcome and analytic approach used. Prospective longitudinal studies incorporating clinically characterized infection history, symptom duration, broader cognitive assessment, and relevant psychological and behavioral factors are needed to clarify whether specific subgroups of young adults experience persistent objective cognitive changes following COVID-19.

## Figures and Tables

**Table 1:** Sample characteristics and cognitive outcomes according to COVID-19 status.

Characteristic	No COVID	COVID Without Consequences	COVID with Consequences
Total n	3945	1676	1861
Age, years, M ± SD	18.5 ± 0.99	18.5 ± 0.95	18.5 ± 0.94
Male, n (%)	1511 (38.3)	730 (43.6)	453 (24.3)
Female, n (%)	2434 (61.7)	946 (56.4)	1408 (75.7)
CTB available, n	3804	1610	1797
CTB missing, %	3.57	3.94	3.44
Choice-response data available, n	3433	1427	1524
Valid latency ≥ 300 ms, n	3425	1423	1513
CTB score, M ± SD	5.37 ± 2.37	5.49 ± 2.33	5.17 ± 2.26
Choice-response accuracy, M ± SD	38.2 ± 3.09	38.4 ± 2.70	38.0 ± 4.05
Correct-response latency, ms, M ± SD	623 ± 144	614 ± 121	623 ± 131
Correct-response latency, ms, median [IQR]	595 [538–671]	590 [537–663]	598 [547–672]

Note. CTB = Corsi Block Tapping Test. Choice-response accuracy is expressed as the number of correct responses out of 40. Latency statistics were calculated for participants with valid mean correct-response latency ≥ 300 ms. IQR = interquartile range.

**Table 2:** Adjusted cognitive differences across COVID-19 groups.

Outcome	COVID Without Consequences vs. No COVID	COVID with Consequences vs. No COVID
CTB, linear mixed model	B = 0.058, 95% CI [−0.077, 0.193], *p* = 0.400	B = −0.146, 95% CI [−0.277, −0.015], *p* = 0.029
CTB, ordinal sensitivity	OR = 1.07, 95% CI [0.96, 1.18], *p* = 0.216	OR = 0.91, 95% CI [0.82, 1.00], *p* = 0.052
Accuracy ^†^	OR = 1.17, 95% CI [1.06, 1.29], *p* = 0.006	OR = 0.97, 95% CI [0.78, 1.20], *p* = 0.729
Latency, primary cleaned analysis ^‡^	−0.10%, 95% CI [−1.18%, 0.99%], *p* = 0.850	−0.46%, 95% CI [−1.52%, 0.61%], *p* = 0.394

Note. Reference group *=* no reported history of COVID-19. CTB linear and latency models were adjusted for age and sex and included a random intercept for university. ^†^ Accuracy estimates are from quasibinomial regression with CR2 cluster-robust inference by university and additionally adjust for log-transformed response latency. ^‡^ Mean correct-response latencies < 300 ms were excluded.

**Table 3:** Association between post-COVID consequences and cognitive performance among previously infected participants.

Cognitive Outcome	n	Effect of Post-COVID Consequences	95% CI	*p*	pFDR
CTB score	3346	B = −0.149	−0.311 to 0.013	0.071	0.188
Choice-response accuracy	2943	OR = 0.80	0.60 to 1.07	0.126	0.188
Correct-response latency	2928	−0.79%	−2.06% to 0.49%	0.224	0.224

Note. Analyses were restricted to participants reporting previous COVID-19. All models were adjusted for acute COVID-19 severity, age, and sex. CTB and latency were analyzed using mixed-effects models with random intercepts for university. Accuracy was analyzed using quasibinomial regression with CR2 cluster-robust inference by university; this infected-only accuracy model was not adjusted for response latency and was not restricted to participants with mean correct-response latency ≥ 300 ms. pFDR values were calculated using the Benjamini–Hochberg procedure across the three cognitive outcomes.

**Table 4:** Sensitivity analysis of CTB performance with additional adjustment for trait anxiety.

Predictor	B	95% CI	*p*
Acute severity: category 2 vs. 1	0.411	−0.174 to 0.996	0.169
Acute severity: category 3 vs. 1	0.531	−0.044 to 1.107	0.070
Acute severity: category 4 vs. 1	0.404	−0.215 to 1.022	0.201
Post-COVID consequences	−0.099	−0.264 to 0.067	0.243
Age	−0.085	−0.169 to −0.001	0.047
Sex	−0.237	−0.419 to −0.056	0.010
Trait anxiety	−0.013	−0.021 to −0.006	<0.001

Note. *n* = 3310; 22 universities. Linear mixed-effects model with a random intercept for university.

## Data Availability

The data presented in this study are available on request from the corresponding author.
